# Role of chaperone-assisted selective autophagy (CASA) in mechanical stress protection of periodontal ligament cells

**DOI:** 10.1007/s00056-021-00358-3

**Published:** 2021-11-04

**Authors:** Corinna Salim, Hannah Muders, Andreas Jäger, Anna Konermann

**Affiliations:** Department of Orthodontics, University Hospital Bonn, Welschnonnenstr. 17, 53111 Bonn, Germany

**Keywords:** Immunohistochemistry, Chaperone–cochaperone complex, Orthodontic tooth movement, Autophagosome, Proteostasis, Immunhistochemie, Chaperon-Cochaperon-Komplex, Kieferorthopädische Zahnbewegung, Autophagosom, Proteostase

## Abstract

**Objective:**

The periodontal ligament (PDL) is exposed to constant mechanical forces potentiated by orthodontic tooth movement (OTM). The aim of our study was to investigate the involvement of chaperone-assisted selective autophagy (CASA) in mechanosensing and cellular adaption to forces in the PDL.

**Materials and methods:**

Human PDL cells were loaded with 2.5, 5, and 10% of static mechanical strain for 24 h in vitro. Untreated cells served as controls. Gene expression of *HSPA8, HSPB8, BAG3, STUB1, SYNPO2* was investigated via RT-qPCR (Quantitative reverse transcription PCR). Western blot evidenced protein expression of these molecules and of Filamin A. In vivo analyses of CASA were performed via immunohistochemistry on teeth with and without OTM.

**Results:**

CASA machinery genes were inherently expressed in PDL cells and exhibited transcriptional induction upon mechanical strain. Protein analyses underlined these findings, even though modulation upon force exertion also demonstrated a decrease for some molecules and loading strengths. In vivo results evidenced again the uniform upregulation of HSPA8, HSPB8, BAG3, STUB1, SYNPO2 and Filamin A in teeth with OTM compared to controls. Experiments generally evidenced a pronounced variability in the expression between donors both on the gene and protein level.

**Conclusions:**

Our study is the first to identify both the expression and functional relevance of CASA in the PDL. The data reflect its probable central role in adequate adaption to forces exerted by OTM and in mechanical stress protection of cells. Deeper knowledge of the CASA pathway will allow better assessment of predisposing factors regarding side effects during mechanical force application that can be used in orthodontic practice.

## Abstract (de)

**Hintergrund:**

Das Parodontalligament (PDL) ist unter physiologischen Bedingungen permanent mechanischen Kräften ausgesetzt, welche durch kieferorthopädische Zahnbewegung (OTM) nochmals potenziert werden. Ziel dieser Studie war die Untersuchung der Rolle von Chaperon-assistierter selektiver Autophagie (CASA) bei der Mechanosensation und der kraftinduzierten Stressadaptation von PDL-Zellen.

**Material und Methoden:**

Humane PDL-Zellen wurden in vitro einer statischen Dehnung von 2,5, 5 und 10 % für 24 h ausgesetzt. Nichtstimulierte Zellen dienten als Kontrolle. Analysiert wurde die Genexpression der CASA-Moleküle *HSPA8, HSPB8, BAG3, STUB1, SYNPO2* via RT-qPCR sowie die Proteinexpression dieser Moleküle und diejenige von Filamin A mittels Western-Blot. In-vivo-Analysen der CASA wurden mit immunohistochemischen Verfahren an Zähnen mit sowie ohne OTM durchgeführt.

**Ergebnisse:**

Alle untersuchten CASA-Moleküle wurden ubiquitär von PDL-Zellen exprimiert und unter mechanischer Belastung hochreguliert. Die Proteinanalysen bestätigten diese Ergebnisse, obwohl eine Modulation bei der Kraftaufwendung für einige Moleküle und Belastungsintensitäten auch einen Rückgang zeigte. Die in vivo Ergebnisse waren deckungsgleich mit den Genanalysen und zeigten eine einheitliche Hochregulation von *HSPA8, HSPB8, BAG3, STUB1, SYNPO2* und Filamin A bei Zähnen mit OTM verglichen mit unbehandelten Kontrollzähnen. Die Experimente zeigten im Allgemeinen eine ausgeprägte Variabilität in der Expression zwischen den Spendern sowohl auf Gen- als auch auf Proteinebene.

**Schlussfolgerungen:**

Unsere Studie ist die erste wissenschaftliche Arbeit, die sowohl die CASA-Moleküle als auch deren funktionelle Relevanz im PDL nachweist. Die Daten zeigen, dass CASA höchstwahrscheinlich eine zentrale Rolle spielt für die adäquate Adaptation an OTM-induzierte Krafteinwirkungen und als protektiver Faktor für Zellen bei mechanischem Stress. Genauere Kenntnisse des CASA-Signalweges werden es ermöglichen, Prädispositionsfaktoren für unerwünschte Wirkungen beim Einsatz mechanischer Belastung künftig besser einzuschätzen.

## Introduction

The periodontal ligament (PDL) represents a mechanically challenged tissue due to its location at the interface between teeth and bone, that has to cope with mechanical forces to maintain cellular viability and homeostasis [2, 10, 16]. The cellular networks are exposed to both physiological strains as well as high amplitude and potentially injurious forces during compression and tension evoked by orthodontic tooth movement (OTM) [14]. Failure to adequately adapt to mechanical load or overload may initiate the pathobiological mechanisms for cellular membrane damage, cell death and subsequent tissue destruction [22]. Furthermore, root resorption as a harmful side effect of OTM might occur if the cellular processes cannot be modulated properly [5]. However, the underlying mechanisms preventing these adverse effects in mechanically active PDL remain to be elucidated. Investigations on other tissues identified a major impact of the processes of protein synthesis and degradation, which seem to be able to orchestrate adaption to mechanical forces, as being important [8, 13, 20, 22]. Here, a fine-tuned balance is considered prerequisite for protein homeostasis of cells, so-called proteostasis [13].

Regarding responses to mechanical forces, chaperone-assisted selective autophagy (CASA) was recently identified as an important tension-induced autophagy pathway coordinating proteostasis in mechanically strained cells and tissues [11, 20]. The molecular chaperones orchestrating this pathway are characterized by a dual role in proteostasis, as they prevent protein aggregation of nonnative clients by folding of damaged specimens, on the one hand, and by participation in degradation and disposal of terminally destructed molecules, on the other hand [20, 22]. These dual modes of action of the chaperones are dependent on regulatory cochaperones that form a chaperone–cochaperone complex with distinct cellular action [20, 22]. Tension-induced CASA, coordinating protein synthesis versus degradation upon mechanical strain, is characterized by client degradation via autophagosome and lysosome formation, and comprises the chaperones HSPA8 and HSPB8, as well as the cochaperones BAG3 and STUB1 [21]. Furthermore, autophagosome formation during CASA is guided by the molecule SYNPO2, which interacts with BAG3 for autophagosome membrane formation around the client-loaded CASA complex [21].

Recent investigations revealed that the CASA complex is also closely related to maintenance of the actin cytoskeleton of cells subjected to mechanical stress [22]. Here, it seems to be responsible for degradation of filamin, which functions as a flexible cross-link of actin and as a mechanosensor, in order to preserve the actin cytoskeleton [3, 15, 17].

Taking into consideration the aspects on cellular mechanotransduction stated above, the hypotheses of our project can be summarized as follows. First, we anticipate verification of the CASA complex components in the PDL, namely the chaperones HSPA8 and HSPB8, as well as the cochaperones BAG3 and STUB1. Second, we will verify involvement of SYNP02 in PDL cells as a potential central factor in the protein degradation machinery activated by pathological mechanical strains in the periodontium guided by autophagosome formation during selective autophagy. As we assume major involvement of the CASA complex in mechanoprotection and maintenance of tissue homeostasis in the PDL, we will investigate these CASA complex components in the PDL particularly exposed to mechanical strain as evoked by OTM. Therefore, we established a standardized cell/tissue system experimental setup based on elastomeric substrates for application of defined mechanical force on living PDL cells with the option to finely adjust tension values. This system allows the cells to be exposed to different tension intensities in order to mimic different stages of orthodontic force. Finally, we hypothesize that protection of the actin cytoskeleton by filamin degradation driven by CASA is a physiological process for maintenance of the proteasome in mechanically strained PDL cells to protect the cytoskeleton and thus withstand tissue degradation. For these objectives of research, we will investigate the impact of different tension values to reveal potential critical force magnitudes during OTM, that might help to prevent unwanted side effects such as external apical root resorption [9].

The aim of this study was to assess the pathophysiological importance of the CASA machinery in the periodontium. Here, we place particular focus on its involvement in OTM and the cellular reaction to mechanical strain.

## Materials and methods

The study was performed according to the ethical principles of the World Medical Association Declaration of Helsinki. Informed consent was obtained from all individual human donors of the experimental material included in the study. The study was independently reviewed and approved by the Ethical Committee of the University of Bonn (reference number 029/08).

### Primary periodontal ligament cell isolation and culture

Human periodontal ligaments (PDL) were utilized to study their potential expression of CASA complex components both on the transcriptional and protein levels. PDL tissues were explanted from the middle third of the root surface of caries-free teeth removed during routine extraction for orthodontic reasons from six periodontally healthy adult male and female donors. Cells were grown in cell culture flasks (T75, CELLSTAR® Greiner BioOne, Kremsmünster, Austria) in N2B27-PDLsf medium (Jäger et al. 2020 [7]) at 37 °C in a humidified 5% CO_2_ atmosphere and passaged after reaching confluence. Medium was supplemented with 1% penicillin–streptomycin (Gibco, Carlsbad, CA, USA) and 1% plasmocin prophylactic (Invivogen, Toulouse, France) until passage 2. From passage 3, both media were used without penicillin–streptomycin or plasmocin prophylactic.

Culture expanded cells were utilized for analyses at passage 3–4. The passaging of cells was performed with StemPro Accutase (Gibco) for 5–10 min at 37 °C and the dissociation was stopped by diluting the enzyme with medium. All conditions were assessed in duplicate.

### Mechanical loading of PDL cells

In order to investigate CASA complex component expression in mechanically loaded PDL cells, static tensile strain was applied to PDL cells cultured to 80% confluence on Bioflex® collagen type I‑coated culture plates with silicone membrane flexible bottom wells (BF-3001C; Flexcell International, Hillsborough, NC, USA). The plates were placed into a strain device (FX-6000T™ Tension System, Flexcell International) provided with a BioFlex baseplate with cylindrical post as loading platform in the dimensions of the flexible-bottom wells. The system comprises a computer-regulated bioreactor using vacuum pressure and positive air pressure to apply a defined, controlled, static deformation to cells growing in monolayer. After cells were seeded into the plates and grown for 24 h before experimentation, continuous cell stretching was performed at 2.5, 5, and 10% [6]. The BioFlex baseplate with the loading stations and the loading posts was placed in an incubator to provide a humidified 5% CO_2_ atmosphere at 37 °C and cells were subjected to mechanical loading for 24 h. Then, plates were removed from the construction and the flexible membranes with the stretched cells were subjected to further experiments as described below. In order to elucidate mechanisms that are provoked by tension-induced CASA, unstretched cells served as controls in each experiment.

### RNA extraction, quality control, and cDNA synthesis

Total messenger ribonucleic acid (mRNA) was isolated and purified from cell lysates using the RNeasy Mini Kit (Qiagen) according to the manufacturer’s protocol. Isolated mRNA was quantified spectrophotometrically (Nanodrop; Thermo Fisher Scientific) and its purity was determined at 260/280 absorbance ratio. mRNA was reverse-transcribed to complementary deoxyribonucleic acid (cDNA) employing the iScript Select cDNA Synthesis Kit (Bio-Rad Laboratories, Hercules, CA, USA). The 20 µl cDNA synthesis reaction oligo(dT) primer mix was prepared according to the manufacturer’s protocol. The maximum available amount of RNA from each sample was used in each synthesis reaction with a cutoff at 1 µg total RNA as the maximum applicable. Synthesis steps were the oligo(dT) primer cDNA reaction at 42 °C for 90 min and the reverse transcriptase inactivation at 85 °C for 5 min performed on an iCycler (Bio-Rad Laboratories).

### RT-qPCR

Quantitative real-time PCR (RT-qPCR) was operated on a StepOnePlus™ RT-PCR System (Thermo Fisher Scientific) with TaqMan® Fast Advanced Master Mix and TaqMan® Gene Ex Assay primers (Thermo Fisher Scientific). The mix was prepared according to the manufacturer’s instruction with 10 ng of the starting cDNA amount. Primers used in this assay were *HSPA8* (hs03044880_gH_FAM), *HSPB8* (hs00205056_m1_FAM), *BAG3* (hs188713_m1_FAM), *STUB1* (hs01071598_g1_FAM), and *SYNPO2* (hs00326493_m1_FAM). Results were standardized to the reference gene *18S *(Hs99999901_s1_VIC), which has emerged as a valid and stable housekeeping gene for PDL cell analyses [19].

Amplification and real-time data acquisition were operated using the following cycle conditions: 2 min at 50 °C, 20 s at 95 °C, followed by 40 cycles with denaturation for 1 min at 95 °C and annealing/extension for 20 s at 60 °C. Negative controls of nuclease-free water were included to obviate DNA contamination in the PCR mix. Melting curve analysis verified the specificity of the PCR products. Data comprise *n* = 6 per condition run in duplicates that were analyzed by the 2^(−∆∆ C(T))^ method according to Pfaffl [18].

### Western blot

For investigation of CASA complex component expression in PDL cells on protein level, western blot analyses were performed for HSPA8, HSPB8, BAG3, STUB1, SYNPO2, and for Filamin A as a molecule modulated via CASA and involved in cytoskeleton protection. PDL cells were maintained as described above. Human muscle cells (kindly provided by Prof. Fürst, Insitute for Cell Biology, University of Bonn, Germany) served as positive control for antibody testing of HSPB8, SYNPO2 and Filamin A. Murine testis (kindly provided by Prof. Schweizer, Insitute of Biochemistry and Molecular Biology, University of Bonn, Germany) served as positive control for antibody testing of HSPA8, STUB1, and BAG3. Whole-cell protein lysates were collected on ice and resuspended in RIPA + cOmplete Mini Protease Inhibitor Cocktail (Roche) sample buffer. Protein concentration was determined by spectrophotometry (BCA protein assay kit; Pierce, Rockford, IL, USA).

In brief, 10 µg of protein was loaded per sample along with prestained protein markers (Precision Plus Protein^TM^ Kaleidoscope^TM^ Standards; Bio-Rad Laboratories, Hercules, CA, USA), electrophoresed on 10% SDS polyacrylamide gels and blotted onto a PVDF membrane (Bio-Rad Laboratories) using a Trans Blot Turbo Transfer System (Bio-Rad Laboratories). The membranes were blocked with 5% milk dissolved in TBS-Tween and incubated at 4 °C overnight on a shaker with anti-human HSPA8 (1:1000; GTX101144; GeneTex, Irvine, CA, USA), anti-human HSPB8 (1:250; orb94695; biorbyt, Cambridge, UK), anti-human BAG3 (1:250; GTX102343; GeneTex), anti-human STUB1 (1:100; GTX109676; GeneTex), anti-human SYNPO2 (1:500; GTX85139; GeneTex), and anti-human Filamin A (1:2000; GTX112939; GeneTex), respectively. Stripped membranes were reprobed with anti-human glyceraldehyde-3-phosphate dehydrogenase (GAPDH; 1:1000; O2/2024, Cell Signaling Technology, Inc., Danvers, MA, USA) as blot loading control. After incubation with horseradish peroxidase (HRP)-conjugated goat anti-rabbit (SouthernBiotech; Birmingham, AL, USA) or rabbit anti-mouse secondary antibodies (Dako) at RT for 1 h on a shaker (dilution 1:1000), blots were developed with SuperSignal West Pico Chemiluminescent Substrate (Pierce) and the ChemiDoc MP Imaging System (BioRad).

Relative quantification of protein bands from western blot films was performed on TIFF file formats changed into grayscale format with ImageJ software (NIH; http://rsb.info.nih.gov/ij/). Quantification reflects the relative amounts of protein as a ratio of the grey value of each protein band relative to the lane’s loading control (GAPDH). After defining the band as region of interest (ROI) for the protein of interest and the loading control, percent deviations were evaluated. For each condition, *n* = 6 was analyzed and applied for mean value calculations.

### Immunohistochemistry

After investigation of the CASA complex components in PDL cells in vitro, we subsequently aimed to verify their potential expression in vivo and to detect contingent differences in their expression patterns in orthodontically moved and unmoved teeth. Teeth extracted for orthodontic reasons due to crowding without OTM served as control specimens. Teeth supporting a fixed rapid palatal expansion appliance were used as moved specimens that had been extracted after rapid maxillary expansion was conducted.

Both paraffin-embedded specimen groups (*n* = 6 per group) were prepared for histological analysis. In brief, all specimens were fixed by immersion in 4% buffered (Sörensen buffer) formaldehyde at RT for at least 1 day and subsequently decalcified in 4.1% disodium ethylenediaminetetraacetic acid (EDTA) solution. After hydration, tissues were dehydrated in an ascending series of ethanol and embedded in paraffin. Serial sagittal sections of 2–3 µm were cut. Selected sections from each specimen were deparaffinized, rehydrated, and rinsed for 10 min in tris-buffered saline (TBS). Endogenous peroxidase was blocked in a methanol/H_2_O_2_ (Merck, Darmstadt, Germany) solution for 10 min in the dark. After being rinsed, sections were incubated with the primary antibodies in a humid chamber. Incubation of primary antibodies was performed with anti-human HSPA8 (1:200; GTX101144; GeneTex), anti-human HSPB8 (1:100; orb94695; biorbyt), anti-human BAG3 (1:100; GTX102396; GeneTex), anti-human STUB1 (1:100; GTX109676; GeneTex), anti-human SYNPO2 (1:200; GTX85139; GeneTex), and anti-human Filamin A (1:100; kindly provided by Prof. Höhfeld, Insitute for Cell Biology, University of Bonn, Germany), respectively.

Chromogen staining of bound antibodies was performed with the anti-mouse/anti-rabbit EnVision+ system (Dako) and diaminobenzodine (DAB; Dako). Mayer’s hematoxylin was used to counterstain the sections. Each experiment was run in triplicate. The validity of the assay was ensured by routine performance of both positive and negative controls for immunohistochemical staining procedures to exclude any artifacts. Negative controls were obtained by replacing the primary antibody with TBS/BSA (bovine serum albumin). Human appendix served as positive control samples to guarantee the specificity for each antibody. Images were taken with a transmitted-light microscope (Axioskop 2, Carl Zeiss Microscopy GmbH, Jena, Germany).

The quality of the original microphotograph of the IHC stainings was double-checked for high resolution and accuracy of the illustration, especially with regard to the coloring to exclude any reduction in quality.

Immunohistochemical quantification of the staining intensities in the samples investigated was performed in order to evaluate and compare the antibody staining intensities in mechanically loaded and control specimens. Image processing was done using QuPath software (https://qupath.github.io). DAB staining intensities were evaluated for each antibody. For each sample, three pictures were taken at × 20 magnification, one of the mesial, one of the distal and one of the apical region of the tooth. In each picture, the PDL was marked as region of interest (ROI) after extracting all interfering structures not belonging to the PDL, e.g., blood vessels. Mean staining intensities were evaluated from the values from *n* = 6 donors with 3 pictures each.

## Statistical analyses

Gene expression changes determined via qRT-PCR and the protein level changes determined via western blot upon exposure to mechanical strain compared to unstimulated cells were analyzed with the one sample t‑test. For statistical evaluations of immunohistochemical stainings, one-way analysis of variance (ANOVA) was applied for comparison between groups, followed by Bonferroni correction. Analytic tests were performed with the GraphPad Prism software (GraphPad Software, San Diego, CA, USA). All values are expressed as mean ± standard error of the mean (SEM) and represent experimental groups with *n* = 6. The level for statistical significance was set at *p* < 0.05.

## Results

### In vitro transcriptional profile of CASA complex-characteristic genes in PDL cells and tension-induced expression changes

**Fig. 1:**
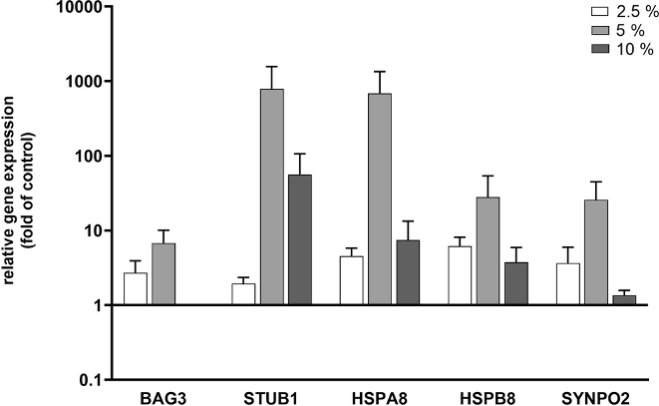
Genetic profile of periodontal ligament (PDL) cells for chaperone-assisted selective autophagy (CASA) complex-characteristic marker genes under different loading intensities. Investigation of expression and transcriptional changes for *BAG3, STUB1, HSPA8, HSPB8*, and *SYNPO2 *in PDL cells. Cultures were exposed to different tension amounts of 2.5, 5, and 10%. Untreated cells served as control. Analyses were performed via *RT-qPCR* (Quantitative reverse transcription PCR). The results were standardized to the reference gene *18S. *Values represent the mean ± standard error of the mean (SEM; *n* = 6) of the relative differential gene expression compared to untreated cells (fold of control). All data were statistically analyzed using one sample t‑test. *P* < 0.05 was considered statistically significant Genprofil von für den CASA(„chaperone-assisted selective autophagy“)-Komplex charakteristischen Markergenen in PDL(Parodontalligament)-Zellen bei unterschiedlichen Belastungsgraden. Untersuchung von Expression und transkriptionellen Veränderungen für *BAG3, STUB1, HSPA8, HSPB8* und *SYNPO2* in PDL-Zellen. Die Kulturen wurden unterschiedlichen Dehnungsbelastungen (2,5, 5 und 10%) ausgesetzt. Als Kontrolle dienten nichtbehandelte Zellen. Die Analysen wurden mittels RT-qPCR durchgeführt, die Ergebnisse wurden auf das Referenzgen *18S* normiert. Die Werte stellen den Mittelwert ± Standardfehler des Mittelwerts (SEM; *n* = 6) der relativen differenziellen Genexpression im Vergleich zu den nichtbehandelten Zellen („fold of control“) dar. Alle Daten wurden mit einem t‑Test für eine Stichprobe statistisch ausgewertet. Als statistisch signifikant wurde *p* < 0,05 festgelegt

Transcriptional analyses were performed to reveal potential verification of CASA in PDL cells, characterized by expression of the chaperones HSPA8 and HSPB8, the cochaperones BAG3 and STUB1 as well as SYNPO2 for autophagosome membrane formation around CASA complexes. Investigations revealed an inherent expression of all molecules investigated, proving that chaperone-assisted proteostasis is a pathway present in the PDL. The relative basal expression in % was highest for HSPA8 with 302.22 ± 240.62, followed by STUB1 with 11.46 ± 9.3. The value for HSPB8 was 0.76 ± 0.52, and lowest basal expression could be detected for SYNPO2 with 0.07 ± 0.04 and BAG3 with 0.08 ± 0.04. As these values already reveal, large variations in the expression level of the genes were detectable between the different donors, which also accounts for the regulatory changes induced by static tensile strain application. CASA complex component expression was markedly upregulated when mechanical loading was exerted on PDL cells, which applied for all strain intensities applied. As evident in Fig. 1, only BAG3 was downregulated to baseline levels at 10% strain, but was upregulated to 2.72 ± 1.21 at 2.5% and to 6.75 ± 3.32 at 5%. HSPB8 and SYNPO2 were only moderately upregulated at 10% with 3.75 ± 2.16 and 1.35 ± 0.23, respectively, compared to the other two lower strain amplitudes. However, STUB1 and HSPA8 were upregulated much more distinctly at 10% with values of 56.22 ± 50.02 and 7.44 ± 5.89 compared to 2.5% with 1.93 ± 0.41 and 4.51 ± 1.30. Most strikingly, all genes investigated featured a marked transcriptional increase for 5% strain, which was most pronounced in the case of STUB1 with 786.02 ± 783.81 and for HSPA8 with 686.07 ± 660.34. This phenomenon was predominantly aroused by one of the PDL cell donors that featured extreme expression changes for all genes investigated at 5% strain. However, even when disregarding this donor, the fact that the values were highest at 5% tensile strain persisted for the genes BAG3 and HSPA8. Staying with the aspect of donor variability, one could observe that all donors used for the studies varied greatly in their responses to the different mechanical loading intensities, which is reflected by the fact that no statistical significances could be detected. However, a general induction of genes upon mechanical stimulation was a uniform and distinct effect seen in the transcriptional analyses.

### Tension-induced protein expression changes of CASA molecules in PDL cells in vitro

**Fig. 2:**
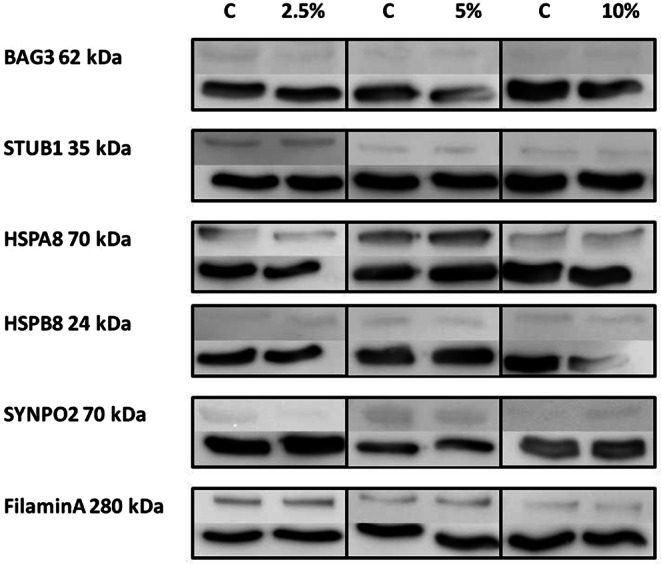
Protein expression of chaperone-assisted selective autophagy (CASA) complex-characteristic molecules in periodontal ligament (PDL) cells at rest and under different loading intensities. Protein expression of BAG3, STUB1, HSPA8, HSPB8, SYNPO2, and Filamin A in PDL cells analyzed via western blot. Cultures were exposed to different amounts of mechanical strain (s) of 2.5, 5, and 10%. The *left lanes* represent the results from the untreated cells serving as control (C), and the *right lanes* show the results for the different amounts of strain. Glyceraldehyde-3-phosphate dehydrogenase (GAPDH, 37 kDa) was used as protein loading control and is presented for each band below the molecule of interest. *kDa* kilodalton. Figures feature representative results from one donor Proteinexpression von für den CASA(„chaperone-assisted selective autophagy“)-Komplex charakteristischen Markergenen in PDL(Parodontalligament)-Zellen in Ruhe und bei unterschiedlichen Belastungsgraden. Proteinexpression von BAG3, STUB1, HSPA8, HSPB8, SYNPO2 und Filamin A in PDL-Zellen, analysiert mittels Western-Blot. Die Kulturen wurden unterschiedlichen mechanischen Belastungen (s) ausgesetzt: 2,5, 5 und 10%. Die *linken Bahnen* zeigen die Ergebnisse der unbehandelten Zellen, die als Kontrolle (C) dienten, die *rechten Bahnen* die Ergebnisse bei den unterschiedlichen Belastungsgraden. Glyceraldehyd-3-Phosphat-Dehydrogenase (GAPDH, 37 kDa) wurde als Proteinladekontrolle verwendet und ist für jede Bande unterhalb des interessierenden Moleküls angegeben. *kDa *Kilodalton. Die Abbildungen zeigen repräsentative Ergebnisse von einem Spender

**Fig. 3:**
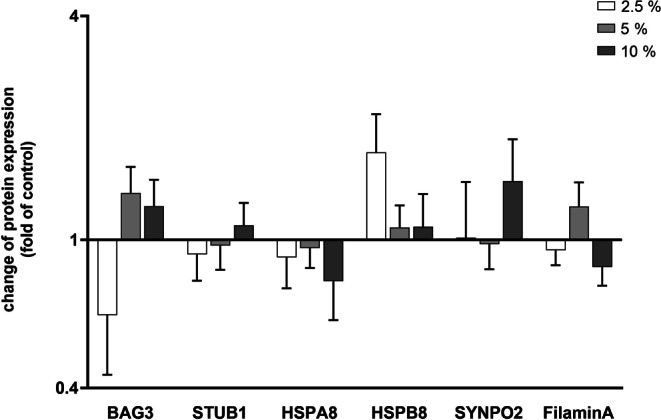
Protein density calculation for chaperone-assisted selective autophagy (CASA) complex-characteristic molecules in periodontal ligament (PDL) cells under different loading intensities compared to cells at rest. Protein density values from western blot analyses were evaluated by measurement of the mean grey values for each blot and normalization to glyceraldehyde-3-phosphate dehydrogenase (GAPDH). Cultures exposed to different amounts mechanical strain of 2.5, 5, and 10% were compared to untreated cells serving as controls. Values represent the mean ± standard error of the mean (SEM, *n* = 6) of the relative differential protein expression compared to untreated cells (fold of control). All data were statistically analyzed using one sample t‑test. *P* < 0.05 was considered statistically significant Berechnung der Proteindichte von für den CASA(„chaperone-assisted selective autophagy“)-Komplex charakteristischen Molekülen in PDL(Parodontalligament)-Zellen bei unterschiedlichen Belastungsgraden im Vergleich zu PDL-Zellen in Ruhe. Proteindichtewerte aus Western-Blot-Analysen wurden durch Messung der mittleren Grauwerte für jeden Blot und Normalisierung auf Glyceraldehyd-3-phosphat-Dehydrogenase (GAPDH) ausgewertet. Kulturen, die unterschiedlichen mechanischen Belastungen (2,5, 5 und 10%) ausgesetzt waren, wurden mit nichtbehandelten Zellen verglichen, die als Kontrollen dienten. Die Werte stellen den Mittelwert ± Standardfehler des Mittelwerts (SEM; *n* = 6) der relativen differenziellen Proteinexpression im Vergleich zu den nichtbehandelten Zellen („fold of control“) dar. Alle Daten wurden mit einem t‑Test für eine Stichprobe statistisch ausgewertet. Als statistisch signifikant wurde *p* < 0,05 festgelegt

After analyzing BAG3, STUB1, HSPA8, HSPB8, and SYNPO2 at the genetic level, we looked at the protein expression of these molecules and investigated whether the pattern is congruent to the transcriptional profile. Furthermore, we included Filamin A in our analyses in order to evaluate the potential process of filamin degradation driven by CASA for protection of the actin cytoskeleton. The findings of the western blot analyses are presented in Fig. 2, showing exemplary results for each molecule investigated, with the control samples (C) on the left side and the tension-stimulated samples on the right side, marked with the corresponding strain intensities, namely 2.5, 5, and 10%. The kilodalton (kDa) information is shown next to the name of the molecule investigated. The band at 70 kDa was assigned to SYNPO2, whereby in the literature the band was reported to be at 117 kD for SYNOP2; thus, protein degradation was likely. First, all molecules of the CASA complex and also Filamin A were basically present in PDL cells on the protein level. Second, inherent expression was again highest for HSPA8 and STUB1, but there was also evidence for a distinct basal expression of Filamin A. HSPB8, SYNPO2, and BAG3 featured a low inherent expression, again consistent with the transcriptional outcomes. Third, the values for all molecules investigated revealed large variations in protein expression levels between the different donors for both the basal expression level and the mode of expression changes upon tensile strain exposure. In Fig. 3, the expression changes upon mechanical stress are presented for all molecules and all strain intensities, featuring the relative fold changes compared to controls. Even though data evaluation showed a consistent downregulation of HSPA8 and upregulation of HSPB8 for all strain intensities and inhomogeneous expression increase and decrease without a clear pattern for BAG3, STUB1, SYNPO2, and Filamin A, the effects were very diverse for the different donors. This is reflected by the large standard deviations, and the resulting lack of statistical significance due to these variations between specimens. Therefore, the data have to be interpreted as trends. Filamin A, which was newly integrated into the analyses in order to evaluate the potential CASA-induced degradation of this mechanosensor, was downregulated 0.94-fold ± 0.08 at low strain exertion of 2.5% and at high strain amplitudes of 10% with 0.85-fold ± 0.09. Contrarily, it was minimally upregulated 1.23-fold ± 0.19 at moderate strain of 5%.

### In vivo expression of CASA complex-characteristic molecules in the PDL of teeth without mechanical loading and teeth upon extensive mechanical force application

Our in vitro investigations uncovered two main findings that can be attributed to the expression and mechanical stress-induced regulation of CASA complex-characteristic molecules in PDL cells. The first aspect is the inherent expression of all target markers, and the second outcome is the pronounced variability in the expression between donors both on the gene and protein level. For this reason, we then wanted to pursue our experimentations in vivo on teeth without and with OTM. The former were teeth extracted for orthodontic reasons due to crowding without OTM serving as controls, and the latter comprised teeth supporting a fixed rapid palatal expansion appliance and being extracted after rapid maxillary expansion was finished.

**Fig. 4:**
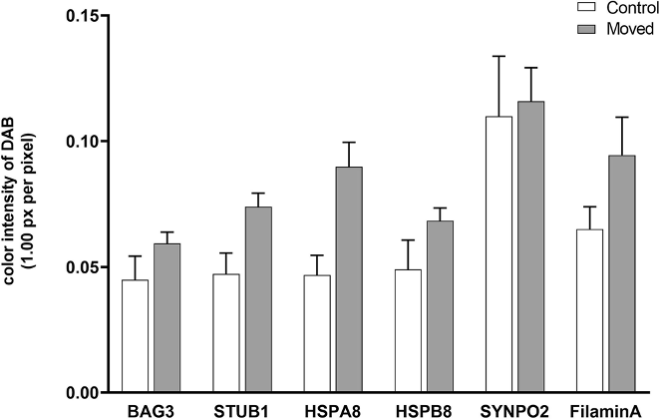
Staining intensity calculation for chaperone-assisted selective autophagy (CASA) complex-characteristic molecules in the periodontal ligament (PDL) of teeth with and without orthodontic tooth movement (OTM). Immunohistochemical quantification of the staining intensities of mechanically loaded (*Moved*) and control (*Control*) specimens for BAG3, STUB1, HSPA8, HSPB8, SYNPO2, and Filamin A. Three regions of interest (ROI) were measured to obtain the mean values for diaminobenzodine (DAB) staining intensities within an image. Values represent the mean ± standard error of the mean (SEM) of *n* = 6 specimens per group. All data were statistically analyzed using one way analysis of variance (ANOVA) and Bonferroni correction. *P* < 0.05 was considered statistically significant Berechnung der Färbeintensität von für den CASA(„chaperone-assisted selective autophagy“)-Komplex charakteristischen Moleküle in PDL(Parodontalligament)-Zellen von Zähnen mit und ohne kieferorthopädische Zahnewegung (OTM). Immunhistochemische Quantifizierung der Färbeintensitäten von mechanisch belasteten Proben (*Moved*) und Kontrollproben (*Control*) für BAG3, STUB1, HSPA8, HSPB8, SYNPO2 und Filamin A. Drei ROI („regions of interest“) innerhalb eines Bildes wurden zur Ermittlung der Mittelwerte für DAB(Diaminobenzodin)-Färbeintensitäten vermessen. Die Werte stellen den Mittelwert ±Standardfehler des Mittelwerts (SEM) von *n* = 6 Proben pro Gruppe dar. Alle Daten wurden mittels Einwegvarianzanalyse (ANOVA) und Bonferroni-Korrektur statistisch ausgewertet. Als statistisch signifikant wurde *p* < 0,05 festgelegt

First, we aimed to verify potential CASA complex-characteristic molecules in vivo*. *Next, we explored the question whether contingent differences can be detected in the expression patterns between orthodontically moved and unmoved teeth. Fig. 4 shows the staining intensity calculations for CASA complex-characteristic molecules in the PDL of teeth with (moved) and without (control) OTM. Results clearly reveal an increase in color intensity for all molecules in OTM teeth compared to controls.

**Fig. 5:**
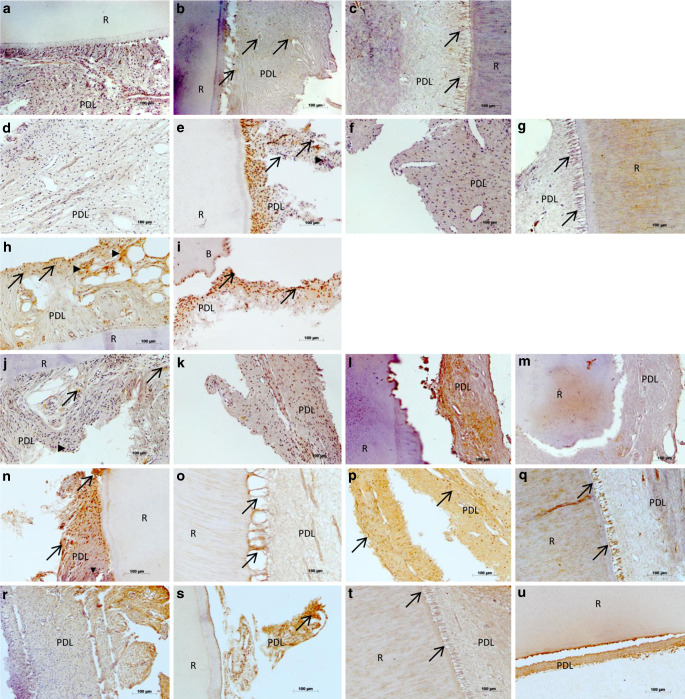
**a**–**u** In vivo verification of chaperone-assisted selective autophagy (CASA) complex-characteristic molecules in the periodontal ligament (PDL) of teeth with or without orthodontic tooth movement (OTM). In vivo protein expression of BAG3 (**a**–**c**), STUB1 (**d**–**g**), HSPA8 (**h**, **i**), HSPB8 (**j**–**m**), SYNPO2 (**n**–**q**), and Filamin A (**r**–**u**) in PDL cells visualized by immunohistochemistry on teeth without OTM extracted due to crowding, and on teeth with OTM that were incorporated in a fixed rapid palatal expansion appliance, followed by extraction. Specifications indicated via *arrows* and *arrowheads* are explained in the “Results” section. *R* Root of the tooth, *B* Bone. Expression intensities were visualized via brown staining with diaminobenzodine (DAB). Magnification × 20 **a**–**u **In-vivo-Nachweis von für den CASA(„chaperone-assisted selective autophagy“)-Komplex charakteristischen Molekülen im Parodontalligament (PDL) von Zähnen mit bzw. ohne kieferorthopädische Zahnbewegung (OTM). In-vivo-Proteinexpression von BAG3 (**a**–**c**), STUB1 (**d**–**g**), HSPA8 (**h**, **i**), HSPB8 (**j**–**m**), SYNPO2 (**n**–**q**) und Filamin A (**r**–**u**) in PDL-Zellen, immunhistochemisch visualisiert an Zähnen ohne OTM, die bei Engstand extrahiert wurden, und an Zähnen mit OTM, die im Rahmen einer schnellen Gaumennahterweiterung in eine festsitzende Apparatur integriert und anschließend extrahiert wurden. Die durch *Pfeile* und *Pfeilspitzen* gekennzeichneten Spezifikationen werden im Abschnitt „Ergebnisse“ erläutert. *R* Zahnwurzel, *B* Knochen. Expressionsstärken wurden durch Braunfärbung mit Diaminobenzodin (DAB) visualisiert. Vergr. 20:1

However, when examining the immunohistochemical outcomes individually for the different donors, analyses revealed marked variations, which can be exemplary seen in Fig. 5. Congruent with the in vitro findings, all molecules investigated were present in the PDL, but with partly large variation in both intensity and localization, which resulted in no statistical significance in the data analysis (Fig. 4).

BAG3 featured a mean staining intensity of 0.04 ± 0.01 for teeth without OTM and of 0.06 ± 0.00 for teeth with OTM. When regarding the tissue samples, BAG3 was insularly expressed in some PDL cells and discretely in extracellular areas of control teeth (Fig. 5a). Fig. 5b, c present teeth with OTM, where the strong force exertion of the fixed rapid palatal expansion appliance is reflected by the extreme stretching of the Sharpey’s fibers at the border to the root (R). The coloring is more pronounced both times compared to controls, but the staining pattern for these two patients is different. In Fig. 5b, several PDL cells are stained evenly distributed over the tissue sample (arrows), whereas in Fig. 5c the dyeing is exclusively localized in the stretched Sharpey’s fibers adjacent to the root surface (arrows).

The expression of STUB1, like BAG3, was low in the PDL at 0.05 ± 0.01 for controls and 0.07 ± 0.01 for moved teeth. This is reflected in Fig. 5d showing a tooth without OTM and almost no staining for STUB1 and in Fig. 5f, g, presenting teeth with OTM with stretched Sharpey’s fibers adjacent to the root surface (arrows), and again almost no or moderate expression. However, illustrating the diversity of the donors, Fig. 5e features an unmoved tooth where STUB1 is strongly expressed in PDL cells (arrowheads) and even in cellular nuclei (arrows).

HSPA8 expression, which was very pronounced in the in vitro experiments, was in vivo almost equivalent to the previous molecules with 0.05 ± 0.01 for controls, while exhibiting higher intensities for moved specimens (0.09 ± 0.01). This is shown in Fig. 5h, i, demonstrating a random, but nevertheless intense expression of HSPA8 in some PDL cells (arrowheads) and cellular nuclei (arrows) of tissues without strain exposure (h), and an intensification with very pronounced staining of almost all nuclei (arrows) in addition to discrete dying of the extracellular space.

The staining intensities for HSPB8 were concordant with those of BAG3 and STUB1, with 0.05 ± 0.01 for teeth without OTM and 0.07 ± 0.00 for teeth with OTM. Fig. 5j shows a control sample with only discrete brown coloration in extracellular spaces (arrowhead) and in single cellular nuclei (arrows). This pattern is clearly intensified in the example shown in Fig. 5k for a tooth with mechanical loading. Fig. 5l, m also feature a tooth with OTM from another donor, presenting the periodontium of the middle third of the root in Fig. 5l and the apical region of the same tooth in Fig. 5m. The staining intensities of HSPB8 were very high in the side area of the tooth, and there was lower intensity staining around the apex, which reveals that there are not only interindividual differences, but that there are also intraindividual differences, depending on the area of interest.

SYNPO2 featured the highest protein expression in the in vivo analyses compared to all other molecules investigated, which is contrary to the results of the in vitro analyses where SYNPO2 expression was very low both on the transcriptional and on protein level. In the in vivo immunohistochemical analyses, staining intensities were 0.11 ± 0.02 for controls and 0.12 ± 0.01 for moved teeth. Fig. 5n demonstrates the strong staining of the entire PDL with both nuclear (arrows) and intracellular (arrowheads) localization in an unmoved tooth, underlining the extent of inherent expression of SYNPO2. Fig. 5o–q represent specimens from three different tooth donors with OTM, uniformly showing the strong expression of the molecule, but again showing various expression patterns between patients. In Fig. 5o and q, SYNPO2 is exclusively concentrated to the stretched Sharpey’s fibers adjacent to the root surface (arrows), whereas in Fig. 5p, the staining extends over the whole tissue with additional nuclear dyeing (arrows).

The highly expressed SYNPO2 was followed by Filamin A with 0.07 ± 0.01 for teeth without OTM and 0.09 ± 0.02 for teeth with OTM. Fig. 5r, s show exemplary tissue staining of unmoved teeth with two different patients, and Fig. 5t, u present specimens with OTM, again from two different patients. These representative pictures again reflect the aforementioned finding that there are strong interindividual differences in the expression of the molecules analyzed. In Fig. 5r, the PDL is completely stained with some focal accumulation of Filamin A but without nuclear enrichment, and in Fig. 5s, the cellular nuclei are also dyed (arrows). In Fig. 5t, the whole PDL is dyed and particularly the intensely stretched Sharpey’s fibers (arrows). In Fig. 5u the staining is extremely strong in the PDL along the surface of the root.

## Discussion

The present study explored the expression and regulation patterns of CASA in PDL in order to assess its potential role in adequate adaption to mechanical load due to OTM. Although previous investigations reported that mechanical signals are transduced into genetic responses by the cells exposed to strain, the associated pathways by which mechanosensing and transcriptional regulation occur are still largely unknown. Our analyses revealed novel findings, as the molecules of the CASA pathway, namely the chaperones HSPA8 and HSPB8, the cochaperones BAG3 and STUB1 as well as SYNPO2 for autophagosome formation can be verified in the PDL in vitro and in vivo*. *Furthermore, these CASA complex components were influenced by tensile strain, indicating an involvement in mechanoprotection of the PDL, as CASA is a tension-induced autophagy pathway [21]. The chaperones HSPA8 and HSPB8 hold key functions in cellular proteostasis, as they maintain a fine-tuned equilibration of protein synthesis and degradation by initiation of the corresponding processes [21]. The dual role of the chaperones plays a major role in maintaining a physiological equilibrium, but its importance increases considerably under stress conditions, where the CASA complex chaperones, connected by the cochaperones BAG3 and STUB1, influence proteostasis in cells subjected to mechanical force. Here, they monitor the interior of the cells for nonnative proteins, and subsequently facilitate folding or degradation [4]. The decision of which path to take with this dual mode of action is driven by the cochaperones. The CASA chaperone–cochaperone complex mediates autophagosome formation enclosing ubiquitylated nonnative clients [12, 20]. STUB1 acts as an ubiquitin ligase and BAG3 exhibits non-overlapping binding sites for both chaperones, thus, providing for their functional interdependence [1]. Autophagosome formation during CASA is also driven by SYNPO2, which simultaneously interacts with BAG3 and with a VPS protein-containing membrane fusion complex, resulting in the formation of an autophagosome membrane around the client-loaded CASA complex [21].

Our in vitro and in vivo expression studies show that CASA complex components were uniformly induced under mechanical loading. The in vitro analyses on the protein level also featured expression reductions for the molecules investigated, except for HSPB8, but the in vivo protein expression experiments clearly showed an induction upon mechanical stimulation. This indicates that in vivo the additional components of the periodontal tissues modulate the overall regulation of the CASA machinery which cannot be exclusively attributed to the strain-dependent increase in PDL cells as seen in vitro.

Transcriptional analyses showed that the magnitude of the applied load did not correspond equally with the magnitude of the gene expression increase, as a smaller force sometimes resulted in a higher upregulation than a larger strain amplitude. This indicates that the different forces utilized in our in vitro study represent physiological force values as routinely used in orthodontics, and that smaller force amplitudes might also induce relevant mechanical stimuli for the cells. Our findings also revealed that the different donors often exhibited completely different expression patterns for the molecules under investigation. This indicates that the different forces utilized in our in vitro study represent physiological force values as routinely used in orthodontics, and that smaller force amplitudes might also induce relevant mechanical stimuli for the cells. Our findings also revealed that the different donors exhibited completely different expression patterns for the molecules under investigation. This phenomenon bears resemblance to external apical root resorption, and the causal relationship between this harmful pathology and OTM remains inconclusive. Here, the individual variations have to be weighted even more than treatment-related factors indicating genetic predisposition and multifactorial etiology, which also seems to account for the initiation of CASA. In addition, our studies focused on the role of Filamin A in the context of CASA and mechanosensing. Filamin A acts as a flexible actin-crosslinker and anchoring protein with the capacity to strongly elongate molecules under the influence of tension, and CASA initiates autophagy of mechanically damaged Filamin A [3, 20]. Current data assume that Filamin A has to be continuously degraded by CASA in mechanically stressed cells to sustain the actin cytoskeleton, and the degraded molecules have to be replaced by newly synthesized Filamin A to maintain the cellular constitution [21]. When regarding our immunohistochemical results, Filamin A was highly concentrated in the mechanically loaded PDL of teeth with OTM, particularly in the stressed and extremely elongated Sharpey’s fibers adjacent to the root surface. In the process of Filamin A replacement, BAG3 holds a dual role by featuring both autophagic disposal of Filamin A in collaboration with SYNPO2, and likewise synthesis of Filamin A under tension [21]. Concordant with the in vivo expression pattern of Filamin A in orthodontically stressed periodontal tissues, BAG3 was likewise concentrated to the stretched Sharpey’s fibers, underlining the close interrelation of these two molecules. The finding that CASA seems to hold a central role in the constant and fine-balanced adjustment of mechanically stressed cells and in resistance against mechanical overload, provides novel insight into the mechanotransduction pathways of the periodontium.

## Conclusion

Based on our research, we were able to show the relevance of the CASA machinery in the periodontium and functionally characterize its impact in mechanical stress protection. The present results provide the basis for greater insight into the precise regulatory pathways underlying both mechanosensing of physiological and pathological forces exerted to the PDL. These data along with future investigations into the involvement of CASA in the regulation of mechanical strain will help us understand the cellular processes initiated by OTM and help us better assess and understand factors associated with side effects due to mechanical force application such as external apical root resorption, which is regularly seen in orthodontic practice.
